# Leadless pacemaker implantation in the region of Bachmann’s bundle: initial results

**DOI:** 10.1093/europace/euag027

**Published:** 2026-02-13

**Authors:** Ashraf Alzahrani, Leena Makhdum, Mohammed Mhanna, Peter Farjo, Paari Dominic, Steven Bailin

**Affiliations:** Division of Cardiovascular Medicine, University of Iowa Health Care, 200 Hawkins Drive, Iowa City, IA 52242, USA; Division of Cardiovascular Medicine, University of Iowa Health Care, 200 Hawkins Drive, Iowa City, IA 52242, USA; Division of Cardiovascular Medicine, University of Iowa Health Care, 200 Hawkins Drive, Iowa City, IA 52242, USA; Division of Cardiovascular Medicine, University of Iowa Health Care, 200 Hawkins Drive, Iowa City, IA 52242, USA; Division of Cardiovascular Medicine, University of Iowa Health Care, 200 Hawkins Drive, Iowa City, IA 52242, USA; Division of Cardiovascular Medicine, University of Iowa Health Care, 200 Hawkins Drive, Iowa City, IA 52242, USA

**Keywords:** Leadless pacemaker, Leadless pacing system, Atrial pacing, Bachmann’s bundle, Conduction system pacing, Physiologic pacing, Pacemaker

## Introduction

Dual-chamber leadless pacemaker technology represents a significant advancement in cardiac rhythm management.^1^ Conventional atrial pacing relies on lead placement in the right atrial appendage (RAA). However, this may result in interatrial conduction delay.^2^ Pacing near Bachmann’s bundle (BB), the primary interatrial conduction tract located at the superior interatrial septum, may offer more physiologic biatrial activation.^2^ However, data on leadless pacing in this region are lacking.

This retrospective series evaluates leadless atrial pacing at the BB region.

## Methods

### Population

Consecutive patients undergoing attempted BB leadless pacemaker implantation between April 2024 and May 2025 were included. The study was IRB-approved with a waiver of consent.

### Procedural description

**Figure 1:**
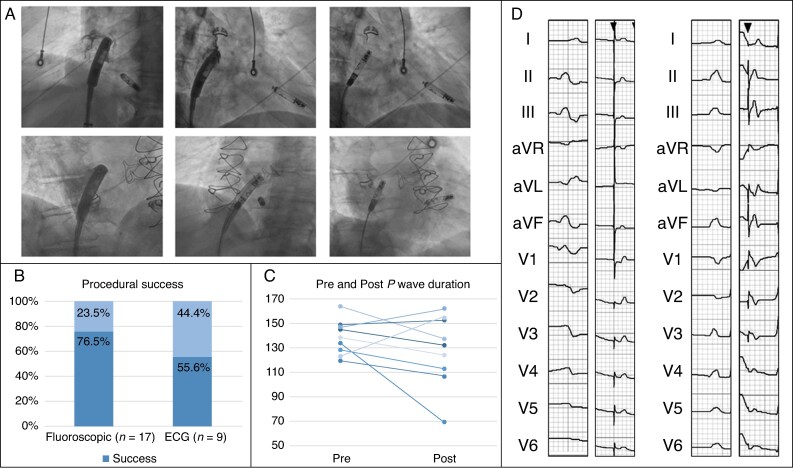
(*A*) Fluoroscopic views demonstrating leadless atrial pacemaker positioning in two patients, shown in left anterior oblique and right anterior oblique projections. (*B*) Bar chart illustrating fluoroscopic implantation success and ECG-defined Bachmann’s bundle capture success. (*C*) Paired line plot comparing pre-implantation and post-implantation P-wave durations on surface ECG. (*D*) Electrocardiograms demonstrating pre- and post-pacemaker implantation P-wave morphology in two patients characterized by upright P-waves in the inferior leads, preservation of native P-wave morphology in lead V1, and shortening of P-wave duration.

Procedures were performed under fluoroscopic guidance via transfemoral venous access using the Aveir™ leadless pacemaker delivery system (Abbott Laboratories, Chicago, IL). To target BB, the device was manipulated in left anterior oblique towards the anterior superior interatrial septum and aligned along the right atrial roof. Right anterior oblique confirmed anterior orientation (*Figure 1A*). Intracardiac contrast verified septal positioning. After electrical testing, the device was fixated, subjected to a tug test, and released.

### Outcomes

Fluoroscopic success was defined as successful deployment of the leadless pacemaker in the BB region, as described by Bailin *et al*.^3^

Electrocardiographic (ECG) success was defined using published criteria^4^:

1. Upright inferior P-waves and biphasic/negative V1
2. Peaked, symmetric inferior P-waves with paced amplitude > sinus
3. P-wave duration shortened by ≥10 ms vs. sinus rhythm

P-wave durations were measured digitally on 12-lead ECG at 50 mm/s. Descriptive statistics were used, with a paired *t*-test used for P-wave comparisons.

## Results

### Baseline characteristics

Seventeen patients underwent attempted BB pacing (mean age 67.2 ± 15.5 years; 53% male; 94% white). Comorbidities included hypertension (71%), diabetes (35%), and coronary disease (29%). Mean left ventricular ejection fraction was 57.7 ± 7%.

### Procedural characteristics

Fluoroscopic success occurred in 76.5% (13/17) (*Figure 1B*). No procedural complications were observed. Reasons for failure included suboptimal positioning, inadequate current of injury, inability to pace after fixation, or helix disruption.

Among successful cases, 84.6% received dual-chamber systems. Indications included sinus node dysfunction (46.2%), complete heart block (38.5%), and second-degree AV block (15.4%). Leadless systems were selected due to prior infection (31%), elevated infection risk (23%), limited life expectancy (23%), or low anticipated pacing burden (23%).

Mean procedure time was 60 ± 13.9 min; mean fluoroscopy time was 9.1 ± 5.6 min; initial mean capture threshold 2.0 ± 0.8 V at 0.6 ± 0.3 ms, sensing 1.9 ± 0.9 mV, and impedance 308.5 ± 67.3 Ω.

### Electrocardiographic characteristics

ECG data were available for nine successful cases. P-wave duration decreased from 138.8 ± 14.1 ms to 128.1 ± 29.1 ms (*P* = 0.1368) (*Figure 1C*). Applying BB criteria, capture was confirmed in 55.6% (5/9). Representative pre- and post-implant ECGs from two cases are shown in *Figure 1D*.

### Follow-up device characteristics

Follow-up was available for nine patients. Mean follow-up was 187.4 ± 116 days, with a median of 168 days (IQR 98–276). Parameters improved over time:

- Capture threshold: 0.9 ± 0.7 V at 0.5 ± 0.3ms
- Sensing amplitude: 3.4 ± 1.3mV
- Impedance: 280 ± 31.6 Ω

Mean atrial pacing percentage was 39%. Estimated battery longevity averaged 5.6 years (RA) and 8.7 years (RV). For dual-chamber devices, i2i throughput was 83% (RV→RA) and 97% (RA→RV).

## Discussion

In this observational study, fluoroscopic-guided leadless atrial pacing near BB was associated with successful positioning in most patients and acceptable pacing parameters. P-wave duration changes were modest, and ECG-confirmed BB capture was observed in a subset of cases. Importantly, these findings should be interpreted in the context of prior studies demonstrating mixed benefits of BB pacing. While physiologic atrial activation remains appealing, evidence suggests that BB stimulation does not consistently translate into meaningful clinical advantage.^2^ Accordingly, the value of this study lies in highlighting the early experience, technical challenges, limitations, and learning points associated with BB region targeting using current leadless platforms.

A key finding of this study is the discrepancy between fluoroscopic success and ECG-confirmed BB capture. As demonstrated by Lustgarten *et al*.,^5^ approximately half of atrial leads positioned fluoroscopically in the BB region engage the conduction tract when electrogram-based criteria are applied. Our ECG-confirmed BB capture rate of 55.6% mirrors this observation, underscoring the limitations of fluoroscopic targeting. Current leadless platforms lack electrogram mapping capability, which contributes to this discordance and limits procedural reproducibility.

Several strategies have been proposed to improve BB engagement. Lustgarten *et al*.^5^ demonstrated that electrogram-guided mapping and pacemapping prior to lead fixation improve true BB capture. More recently, Hollis *et al*.^6^ reported successful leadless BB region pacing using intracardiac echocardiography, highlighting the role of imaging to refine engagement. Technically, precise septal–roof targeting, controlled device orientation, and stable tissue contact are critical for reliable BB engagement.

From a broader perspective, this early observational experience fits within the historical evolution of cardiac pacing towards leadless and increasingly physiologic strategies over the past two decades.^7^ Within this trajectory, emerging reports of leadless left bundle branch area pacing demonstrate the feasibility of leadless ventricular conduction system pacing.^8^ In parallel, anatomic and modelling studies continue to refine optimal and safest atrial implant sites, underscoring the ongoing evolution of leadless pacing technology.^9^

Safety considerations of BB pacing include anatomic variability, difficulty achieving stable fixation on the atrial roof, protrusion across a patent foramen ovale, poor pacing parameters in fibrotic septa, and rare instances of aortic erosion.^10^ Existing studies, however, show similar complication rates between BB and RAA pacing.^2^

Our study has limitations, including the single-centre design, small sample size, retrospective methodology, limited follow-up, incomplete availability of follow-up ECG data, and absence of electrogram-based BB confirmation. In addition, ECG-derived *P*-wave measurements are operator-dependent, and the observed discordance between fluoroscopic positioning and ECG-confirmed BB capture may limit procedural reproducibility. Lastly, clinical outcomes such as AF burden were not assessed.

## Conclusion

Leadless atrial pacing near BB was associated with acceptable procedural performance using an anatomy-based fluoroscopic approach in this early experience. Larger, prospective studies are needed to define reproducibility and clinical impact.

## Data Availability

The data underlying this article cannot be shared publicly due to the privacy of individuals who participated in the study. De-identified data will be shared on reasonable request to the corresponding authors.
